# The effect of BACE1-AS on β-amyloid generation by regulating BACE1 mRNA expression

**DOI:** 10.1186/s12867-019-0140-0

**Published:** 2019-10-01

**Authors:** Fan Li, Yun Wang, Hui Yang, Yingying Xu, Xiaoyan Zhou, Xiao Zhang, Zhaohong Xie, Jianzhong Bi

**Affiliations:** Department of Neurology, The Second Hospital of Shandong University, Shandong University, 247 North Park Avenue, Jinan, 250033 China

**Keywords:** Alzheimer’s disease, lncRNA, BACE1-AS, Aβ

## Abstract

**Background:**

The BACE1 antisense transcript (BACE1-AS) is a conserved long noncoding RNA (lncRNA). The level of BACE1-AS is significantly increased and the level of the BACE1 mRNA is slightly increased in subjects with AD. BACE1-AS exerts a significant moderating effect on the expression of the BACE1 mRNA and promotes the formation of Aβ. After the administration of Aβ_1-42_ to SH-SY5Y cells and C57/BL6J mice, we detected the expression of BACE1-AS, BACE1 mRNA, and BACE1 protein, as well as the concentration of Aβ_1-40_. Then, we silenced the expression of BACE1-AS in SH-SY5Y and 20E2 cells using siRNAs targeting BACE1-AS and detected its effects on the levels of the BACE1 mRNA and BACE1 protein and Aβ_1-40_ generation.

**Results:**

The administration of Aβ_1-42_ increased the expression of BACE1-AS, BACE1 mRNA and protein, as well as the concentration of Aβ_1-40_ in SH-SY5Y cells and the brains of C57BL/6J mice. Pretreatment with the BACE1-AS siRNA inhibited the effect of Aβ_1-42_ on increasing the expression of BACE1-AS and BACE1, as well as the generation of Aβ.

**Conclusions:**

The mechanism by which exogenous Aβ_1-42_ induces BACE1 expression and Aβ generation is mediated by BACE1-AS. BACE1-AS is involved in the mechanism regulating BACE1 expression and Aβ generation in APPsw transgenic cells.

## Background

Alzheimer’s disease (AD) is a central nervous system disease occurring in senile and presenile phases that is characterized by progressive cognitive and behavioral impairments. Currently, a specific treatment for AD is unavailable. Therefore, further study of the etiology and pathogenesis of AD that aims to identify new ways to prevent and cure AD may resolve this major problem [1].

The amyloid cascade hypothesis plays a key role in the study of the pathogenesis of AD. The deposition of β amyloid (Aβ) is considered to lead to the dysfunction and death of neurons in the brain. Aβ generation from APP is mediated by proteases and is currently the most well-defined part of this hypothesis. β-Site APP cleaving enzyme 1 (BACE1) is an endogenous beta secretase mediating the cleavage of APP [2]. Aβ is not detected in BACE1 gene knockout mice [3], and the injection of a small interfering RNA targeting BACE1 into the mouse ventricle reduces the Aβ deposition and cognitive decline in APP transgenic mice [4].

A noncoding RNA (ncRNA) is an RNA that is not translated into a protein but has biological functions. A long noncoding RNA (lncRNA) refers to a noncoding RNA of more than 400 nucleotides in length and includes the long intergenic noncoding RNA (lincRNA), natural antisense transcript (NAT) and noncoding RNA repeats, etc. [5]. Some lncRNAs directly positively or negatively regulate the expression of adjacent genes, whereas others regulates the activity of proteins by interacting with proteins [6]. Although many types of lncRNAs are specifically expressed in the nervous system, a limited number of reports have described the abnormal expression of natural antisense transcripts in subjects with AD, and their functions and mechanisms of regulation remain to be fully clarified [7, 8]. NATs are specifically expressed in the central nervous system and are considered important contributors to the mechanisms regulating neural function [9, 10].

BACE1 antisense transcript (BACE1-AS) is a conserved 2 KB noncoding antisense transcript that is transcribed from the antisense strand of the BACE1 gene locus on chromosome 11 (11q23.3) [11]. According to the results of the study by Faghihi MA, the expression of BACE1-AS and BACE1 mRNA is upregulated and the Aβ_1-42_ content is markedly increased in the brains of patients with AD and APP-tg19959 APP transgenic mice, suggesting that the abnormal activation of BACE1-AS is involved in the pathogenesis of AD [12]. In SH-SY5Y cells and HEK293T cells lacking BACE1-AS, levels of the BACE1 mRNA and protein, as well as Aβ_1-40_ and Aβ_1-42_ are significantly decreased, indicating that BACE1-AS exerts a significant regulatory effect on the expression of the BACE1 mRNA [12].

Exogenous Aβ_1-42_ increases BACE1-AS expression in neuronal cells in vitro. Further research showed that BACE1-AS increases the stability of the BACE1 mRNA in cells by directly interacting with the BACE1 mRNA, resulting in increased levels of the BACE1 protein and increased generation of Aβ_1-40_ and Aβ_1-42_ from APP through the β secretase pathway. The increased Aβ levels in turn increased the expression of BACE1-AS, thus forming a positive feedback loop. BACE1-AS and microRNA mir-485-5p, which inhibits BACE1 mRNA expression, have common binding sites within the BACE1 mRNA; therefore, they have a mutually antagonistic relationship in regulating the expression of the BACE1 mRNA [13].

The addition of a BACE1-AS siRNA into the culture medium of SH-SY5Y cells and HEK293T cells cultured in vitro inhibits the expression of BACE1-AS and BACE1 mRNA and reduces the level of the BACE1 protein. The addition of siRNAs targeting the BACE1 mRNA only inhibits the expression of the BACE1 mRNA and reduces the level of the BACE1 protein, but does not exert a significant effect on BACE1-AS expression. After BACE1-AS knockout, the number of cells producing Aβ_1-40_ and Aβ_1-42_ decreases significantly. Based on these results, BACE1-AS exerts a significant regulatory effect on the expression of the BACE1 mRNA [12]. After adding 1 μM Aβ_1-42_ to the cell culture medium, an increase in BACE1-AS expression is observed within 2 h, and the BACE1 protein content increases in 12 h. Thus, Aβ_1-42_ increases the stability of the BACE1 mRNA by increasing BACE1-AS expression and subsequently increasing the synthesis of the BACE1 protein, thus promoting the generation of Aβ_1-40_ and Aβ_1-42_ and the positive feedback loop to generate Aβ [12].

As described above, BACE1-AS plays an important role in the pathogenesis of AD, and previous studies have partially elucidated the regulatory mechanism. However, researchers have not clearly determined whether the expression of BACE1-AS in the mouse brain is increased by Aβ_1-42_, rather than other effects of APP gene transfer. We used siRNAs targeting BACE1-AS to inhibit BACE1-AS expression and observed its effect on the levels of the BACE1 mRNA and protein, as well as Aβ_1-40_ generation, to address this question. Next, we observed the expression of BACE1-AS, BACE1 mRNA and Aβ_1-40_ levels in the brains of C57/BL6J mice after directly injecting Aβ_1-42_ into the hippocampus using a stereotactic technique to determine the effect of Aβ_1-42_ on the expression of BACE1-AS in vivo.

## Results

### Exogenous Aβ_1-42_ increases the expression of BACE1-AS and BACE1 and the generation of Aβ in SH-SY5Y cells

**Fig. 1:**
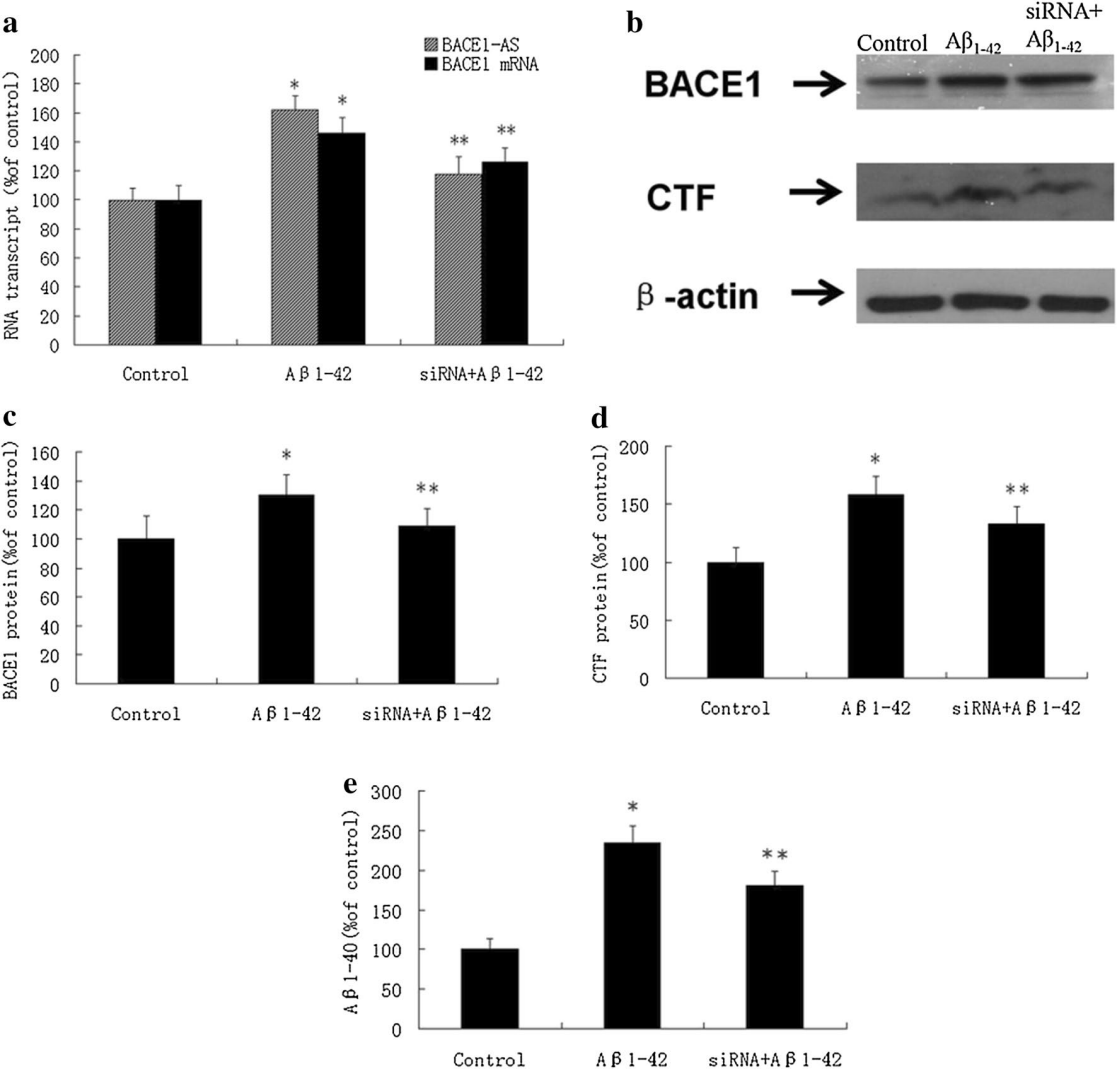
Exogenous Aβ_1-42_ promotes Aβ production by increasing BACE1-AS expression in SH-SY5Y cells. After treating SH-SY5Y cells with Aβ_1-42_, the expression of BACEl-AS and BACEl were significantly elevated (*P <  0.05). After the pretreatment with BACE1-AS siRNA for 24 h followed by treatment with Aβ_1-42_, the expression of BACEl-AS and BACE1 mRNA significantly decreased (*P < 0.05) (**a**). After treating SH-SY5Y cells with Aβ_1-42_ , the expression of BACEl and C1F protein (**b**, **c**), and also the concentration of Aβ_1-40_ (**d**) in extracellular fluid were significantly elevated (*P *<* 0.05). After the pretreatment with BACEl-AS siRNA for 24 h followed by treatment with Aβ_1-42_ for 2 h, the expression of BACEl and CTF protein (**b**, **c**), and also the concentration of Aβ_1-40_ (**d**) significantly decreased (*P < 0.05)

After treating SH-SY5Y cells with Aβ_1-42_ (1 μM) for 2 h, the expression of BACE1-AS and BACE1 was significantly elevated compared with the control group that was treated with an equal amount solvent lacking Aβ_1-42_ (Fig. 1a). Levels of the BACE1 and C-terminal fragment (CTF) proteins were significantly increased (Fig. 1b–d). Aβ_1-40_ concentrations were significantly increased 24 h after the addition of Aβ_1-42_ (1 μM) (Fig. 1e).

### The effect of silencing BACE1-AS expression with siRNAs on the expression of BACE1 and the generation of Aβ_1-40_ and Aβ_1-42_ in SH-SY5Y cells

We pretreated SH-SY5Y cells with three different BACE1-AS siRNAs (siRNA_a, siRNA_b, and siRNA_c, sequences shown in Table 1) for 12, 24 or 48 h before adding Aβ_1-42_ (1 μM). After a 2 h incubation, intracellular RNA was collected and extracted from each dish, and BACE1-AS expression was detected using the fluorescent TaqMan probe and quantitative PCR. The interference rate was 60–70% 24 h after treatment with the three siRNAs. Therefore, we chose a 24 h pretreatment with the BACE1-AS siRNA for subsequent experiments. After pretreatment with the BACE1-AS siRNA, the expression of BACE1-AS and BACE1 mRNA was significantly decreased (Fig. 1a). Levels of the BACE1 and CTF proteins were significantly reduced (Fig. 1b–d), and the concentration of Aβ_1-40_ in the supernatant was substantially decreased (Fig. 1e).

**Table 1:** Oligonucleotide sequences used for Taqman PCR and siRNA sequences

Primer name	Application	Sequence or assay ID
1. Human BACE1-AS–F	Real-time PCR	GAAGGGTCTAAGTGCAGACATCTT
2. Human BACE1-AS–R	Real-time PCR	AGGGAGGCGGTGAGAGT
3. Human BACE1-AS–P	Real-time PCR	ACATTCTTCAGCAACAGCC
4. Mouse BACE1-AS–F	Real-time PCR	GTAGGCAGGGAAGCTAGTACTGA
5. Mouse BACE1-AS–R	Real-time PCR	AGAGGCTTGCAGTCCAGTTC
6. Mouse BACE1-AS–P	Real-time PCR	CCTGGAAGGAGAAACAG
7. Human BACE1–F	Real-time PCR	Taqman(Assay ID: Hs00201573_m1)
8. Human BACE1–R	Real-time PCR	Taqman(Assay ID: Hs00201573_m1)
9. Human BACE1–P	Real-time PCR	Taqman(Assay ID: Hs00201573_m1)
10. Mouse BACE1–F	Real-time PCR	Taqman(Assay ID: Mm00478664_m1)
11. Mouse BACE1–R	Real-time PCR	Taqman(Assay ID: Mm00478664_m1)
12. Mouse BACE1–P	Real-time PCR	Taqman(Assay ID: Mm00478664_m1)
13. Human BACE1-AS siRNA_a	siRNA	CCCTCTGACACTGTACCATCTCTTT
14. Human BACE1-AS siRNA_b	siRNA	AGAAGGGTCTAAGTGCAGACATCTG
15. Human BACE1-AS siRNA_c	siRNA	CCAGAAGAGAAAGGGCACT

### Exogenous Aβ_1-42_ induces BACE1-AS and BACE1 expression and Aβ generation in the brains of C57BL/6J mice

**Fig. 2:**
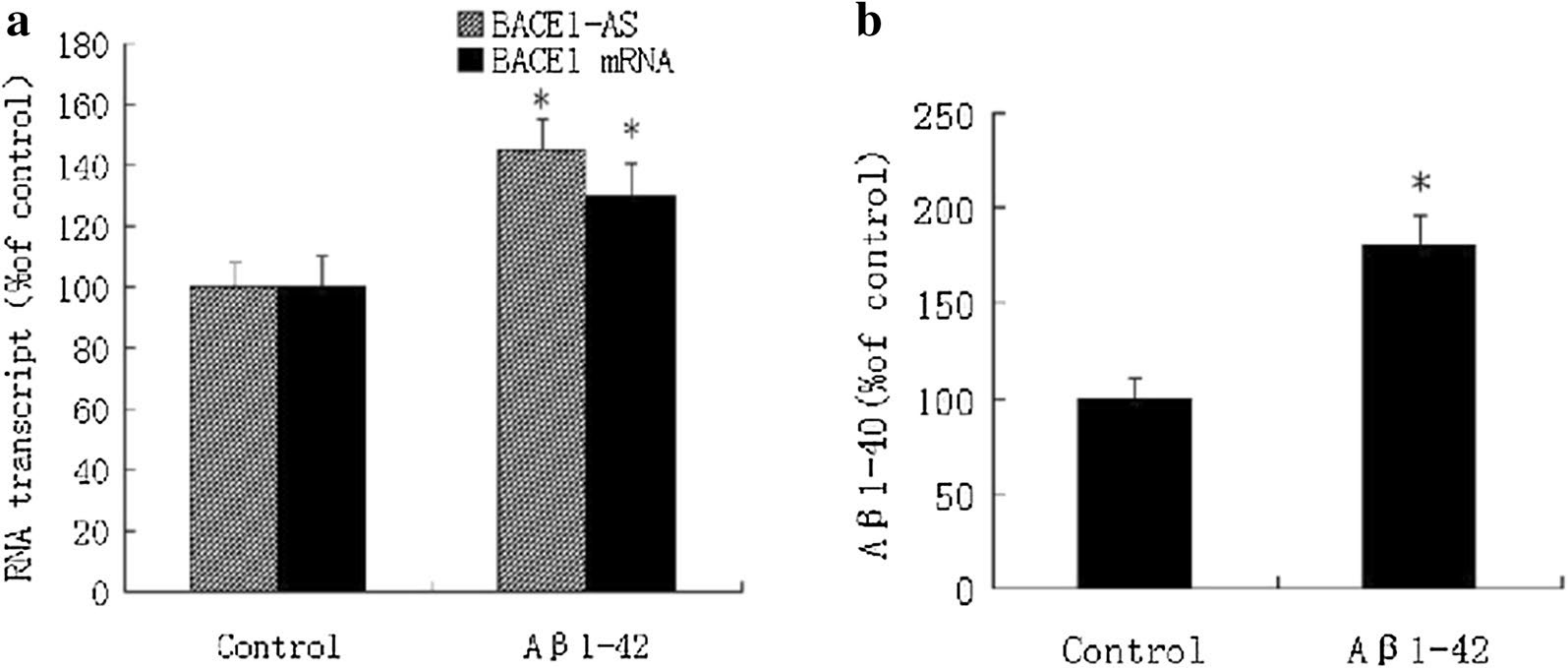
Exogenous Aβ_1-42_ induces BACE1-AS and BACE1 expression and Aβ generation in the brains of C57BL/6J mice. Three days after injection of Aβ_1-42_ into the CA1 region of left side hippocampus of the mouse using experimental group using stereotactic technique, the expression of BACEl-AS and BACEl mRNA increased in the hippocampus on the injected side (*P < 0. 05 vs. control group) (**a**), and the content of Aβ_1-40_ raised compared with the control group (*P < 0.05 vs. control group) (**b**)

We randomly divided 3-month-old C57BL/6J mice into an experimental group and control group comprising 7 mice each to determine the effect of Aβ_1-42_ on BACE1-AS and BACE1 expression and Aβ production in C57BL/6J mice in vivo. Two microliters of a solution containing 1 μg of Aβ_1-42_ were injected into the CA1 region of the left hippocampus in each mouse of the experimental group using a stereotactic technique, while an equal amount solvent was injected into the same region of the mice in the control group. Three days after the injection of Aβ_1-42_, the expression of BACE1-AS and BACE1 mRNA increased in the hippocampus on the injected side (Fig. 2a), and the Aβ_1-40_ concentration was increased compared with the control group (Fig. 2b).

### The effect of transfection of the APPsw gene on BACE1-AS and BACE1 expression and Aβ generation in 20E2 cells

**Fig. 3:**
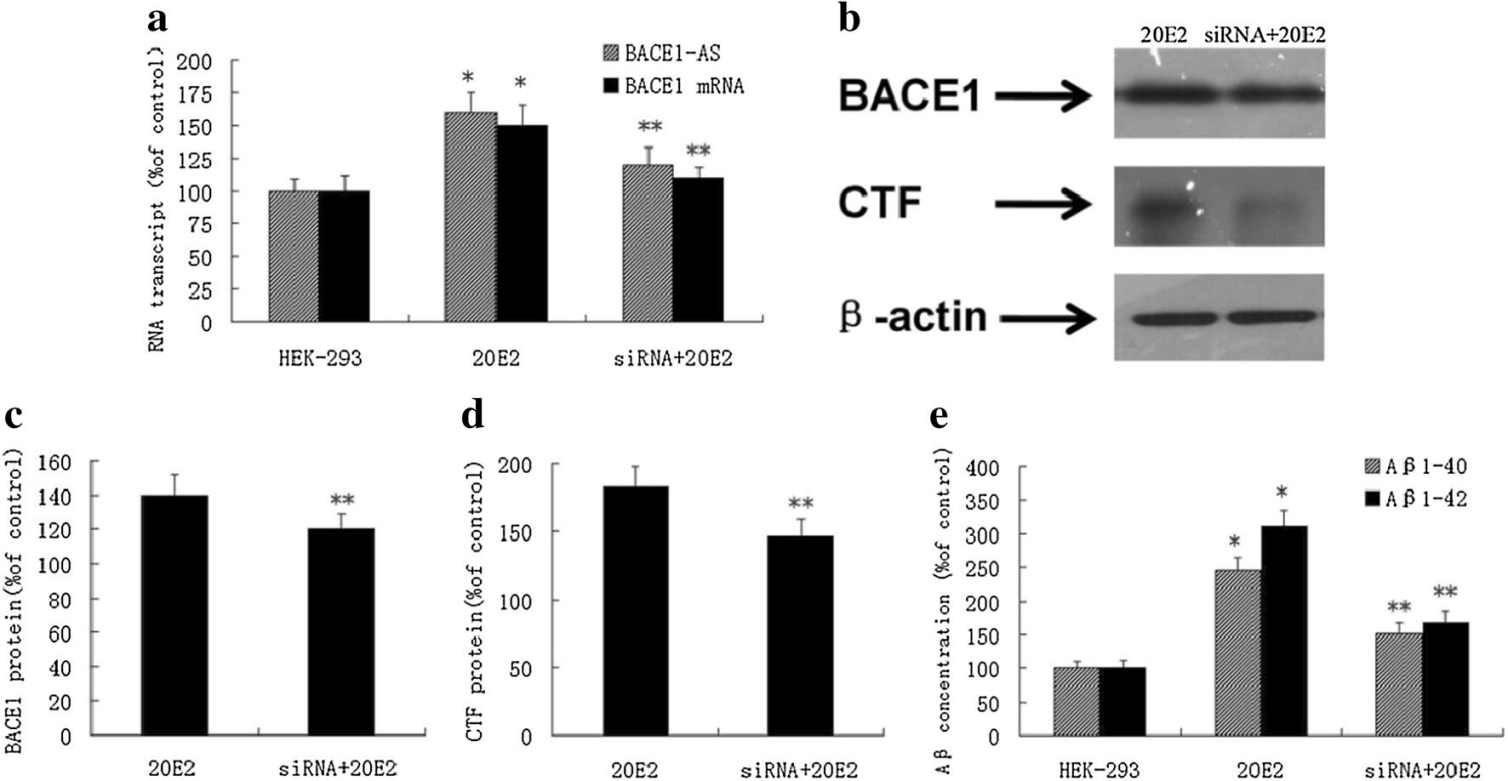
Effect of BACE1-AS silencing with siRNAs on BACE1 expression and Aβ_1-40_ and Aβ_1-42_ production in 20E2 cells

The 20E2 cells are HEK293 cells that carry and express the Swedish mutant APP695 (APPsw) gene. We used 20E2 cells to study the effect of transfecting the APPsw gene on BACE1-AS and BACE1 expression and Aβ_1-40_ and Aβ_1-42_ generation in cells. Intracellular RNA was extracted and the supernatant was collected after 24 h of culture. The expression of intracellular BACE1-AS and BACE1 mRNA was detected with the TaqMan probe and fluorescent quantitative PCR, and the concentrations of Aβ_1-40_ and Aβ_1-42_ in the supernatant were detected with ELISAs. After transfecting the APPsw gene, the expression of BACE1-AS and BACE1 mRNA was significantly increased in cells (Fig. 3a), and significantly higher concentrations of Aβ_1-40_ and Aβ_1-42_ were detected in supernatants from 20E2 cells than in supernatants from HEK293 cells (Fig. 3e).

### Effects of BACE1-AS silencing with siRNAs on BACE1 expression and Aβ_1-40_ and Aβ_1-42_ production in 20E2 cells

We treated 20E2 cells with the BACE1-AS siRNA for 24 h to study the effects of BACE1-AS silencing with siRNAs on BACE1 expression and Aβ_1-40_ and Aβ_1-42_ generation in APPsw transgenic 20E2 cells; the interference rate was 60–70%. After treatment with the BACE1-AS siRNA, the expression of BACE1-AS and BACE1 mRNA was significantly decreased in 20E2 cells (Fig. 3a). Levels of the BACE1 and CTF proteins were significantly reduced (Fig. 3b–d), and the concentrations of Aβ_1-40_ in the supernatant were substantially decreased (Fig. 3e).

## Discussion

AD is a devastating neurodegenerative disease that causes progressive damage to neurons, and no curative treatment for AD is available because the etiology of AD is poorly understood. Epigenetic mechanisms have recently been reported to be already dysregulated during the early stages of disease progression [14].

Based on accumulating evidence, lncRNAs are not transcriptional noise but play crucial roles in regulating gene expression at the epigenetic, transcriptional, and posttranscriptional levels, and the dysregulation of lncRNAs is associated with disease development [15]. More recently, lncRNAs have been implicated in neurodegenerative diseases, including AD, where the role of the BACE1-AS lncRNA has been widely defined [16, 17].

BACE1-AS is a conserved long noncoding antisense transcript, and several studies have reported that BACE1-AS is a crucial enzyme in AD pathophysiology [18, 19]. BACE1-AS expression is elevated in subjects with AD and drives the rapid feed-forward regulation of β-secretase, suggesting that it is critical for AD development [20]. Plasma BACE1 levels are increased in patients with AD, indicating that BACE1 may be a potential candidate biomarker to predict AD [21]. The expression of the lncRNA BACE1-AS is upregulated in peripheral blood samples and hippocampi from an AD animal model. Knockdown of BACE1-AS with siRNAs increases the proliferation of primary hippocampal neurons. Knockdown of BACE1-AS with a lentivirus improves the memory and learning behaviors of SAMP8 mice and inhibits BACE1 and amyloid precursor protein production and phosphorylation of the tau protein in the hippocampus [22].

BACE1-AS expression is upregulated in the frontal cortex, hippocampus, and olfactory cortex in the brains of patients with AD, suggesting that BACE1-AS might participate in the pathogenesis of AD. BACE1-AS binds to the BACE1 mRNA to increase its stability, thereby promoting the synthesis of the BACE1 protein and further increasing the production of Aβ. BACE1 mRNA expression is controlled by a regulatory noncoding RNA that may drive AD-associated pathophysiology. Upon exposure to various cell stressors, including Aβ_1-42_, BACE1-AS expression increases, subsequently increasing BACE1 mRNA stability and generating additional Aβ_1-42_ through a posttranscriptional feed-forward mechanism [12]. BACE1-AS and the microRNA mir-485-5p, which inhibits the expression of the BACE1 mRNA, have common binding sites in the BACE1 mRNA; therefore, they have a mutually antagonistic relationship in regulating the expression of the BACE1 mRNA [13]. BACE1-AS levels are associated with HuD, an RNA-binding protein that is primarily expressed in neurons and implicated in learning and memory. Higher BACE1-AS levels are observed in the brains of HuD-overexpressing mice [23].

Researchers have not clearly determined whether exogenous Aβ_1-42_ induces BACE1-AS and BACE1 expression and Aβ generation in vivo, or whether the expression of BACE1-AS in the brains of APP transgenic mice is increased by Aβ_1-42_, rather than other effects of APP gene transfer.

Based on our in vitro experiments, after culturing SH-SY5Y cells with exogenous Aβ_1-42_ (1 μM) for 2 h, levels of BACE1-AS, the BACE1 mRNA, and the BACE1 and CTF proteins were significantly increased. Additionally, the concentration of Aβ_1-40_ in the supernatant increased significantly 24 h after treatment. These results are consistent with the findings reported by Faghihi MA et al. [12].

Pretreatment with the BACE1-AS siRNA significantly inhibited the Aβ_1-42_-induced increase in the expression of BACE1-AS and BACE1 in SH-SY5Y cells. After pretreatment with the siRNA, levels of BACE1-AS, the BACE1 mRNA, and the BACE1 and CTF proteins were significantly decreased in cells, and the concentration of Aβ_1-40_ in the supernatant was noticeable decreased. Thus, BACE1-AS silencing inhibited the effect of exogenous Aβ_1-42_ on inducing BACE1-AS and BACE1 expression and Aβ generation, subsequently interrupting the positive feedback loop by which Aβ increases BACE1 and Aβ levels by activating BACE1-AS and increasing the stability of the BACE1 mRNA.

Meanwhile, we used stereotactic injections to observe the effect of exogenous Aβ_1-42_ on BACE1-AS and BACE1 expression and Aβ production in the brains of C57BL/6J mice. Exogenous Aβ_1-42_ significantly increased the expression of BACE1-AS and the BACE1 mRNA, as well as the Aβ_1-40_ levels in the hippocampus on the injected side 3 days after the intervention compared with the control group. Based on these results, the stereotactic injection of exogenous Aβ_1-42_ induced BACE1-AS and BACE1 expression and Aβ generation, suggesting that Aβ1-42 increased the expression of BACE1-AS and BACE1 and Aβ generation in vivo, thus forming the positive feedback loop of Aβ generation. Our results are consistent with the findings reported by Zhang et al. [22]. This finding might also be one of the molecular mechanisms underlying the deteriorating pathological physiological processes in the brains of patients with AD, providing a possible new target for the studies of the etiology and treatment of AD in human subjects.

As shown in the present study, the intracellular expression of BACE1-AS and BACE1 mRNA, and the concentrations of Aβ_1-40_ and Aβ_1-42_ in the supernatant were increased in 20E2 cells transfected with the APPsw gene compared with HEK293 cells. After silencing BACE1-AS with siRNAs, levels of the BACE1 mRNA and BACE1 and CTF proteins in 20E2 cells were significantly decreased, and the concentrations of Aβ_1-40_ and Aβ_1-42_ in the supernatant were also significantly decreased compared with the untreated group. Thus, BACE1-AS expression is abnormally induced in APPsw transgenic cells, and the silencing of BACE1-AS inhibits the positive feedback cycle produced by endogenous Aβ_1-42_ that induces the abnormal expression of BACE1-AS, thus reducing Aβ production. This study provides a good cell model and research platform for further exploration of treatment methods employing BACE1-AS in the studies of treatment strategies for AD.

## Conclusions

The mechanism by which exogenous Aβ_1-42_ induces BACE1 expression and Aβ generation is mediated by BACE1-AS. BACE1-AS is involved in the mechanism regulating BACE1 expression and Aβ generation in APPsw transgenic cells.

## Methods

### Cell culture

Human SH-SY5Y neuroblastoma cells and 20E2 cells (Purchased from Cell Resource Center, Chinese Academy of Medical Science, Peking Union Medical College, Beijing, China) were cultured in Dulbecco’s Modified Eagle’s Medium (DMEM) (HyClone, Los Angeles, California, USA) supplemented with 1× nonessential amino acids, 1 mM sodium pyruvate, 1.5 g/L sodium bicarbonate, and 10% fetal bovine serum and incubated in a 5% CO_2_ incubator at 37 °C. Culture media were refreshed every second day. All assays were repeated at least three times.

### RNA interference

**Fig. 4:**
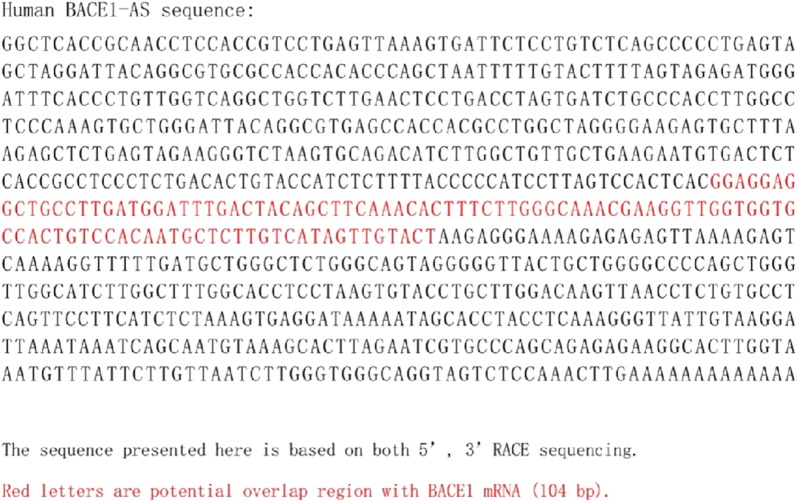
Oligonucleotide sequences for human BACE1-AS [12]

**Fig. 5:**
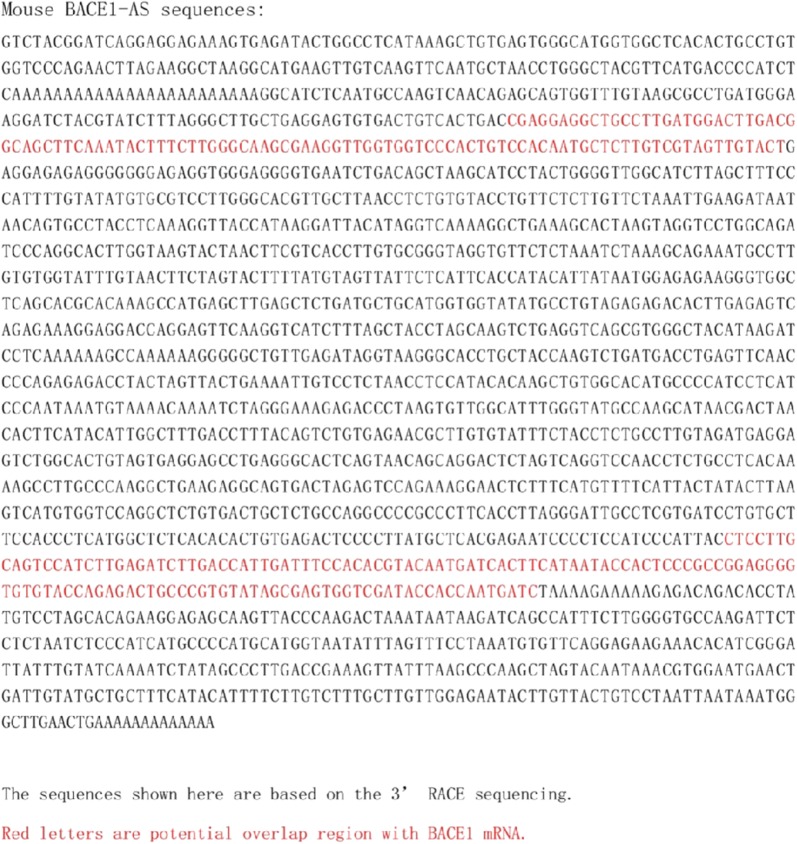
Oligonucleotide sequences for mouse BACE1-AS [12]

The BACE1-AS siRNA was purchased from Gene Pharma Company (Shanghai, China) (oligonucleotide sequences for human and mouse BACE1-AS are shown in Figs. 4 and 5, respectively, and BACE1-AS siRNA sequences are defined in Table 1). Briefly, 5 μL of siRNA duplexes and 5 μL of the Lipofectamine 2000TM liposome reagent (Invitrogen, Carlsbad, California, USA) were added to 500 μL of serum-free Opti-MEM without antibiotics (Invitrogen, Invitrogen, Carlsbad, California, USA) and mixed gently. The 40 nM transfection mixture was mixed with 90 μL of transfection buffer, 6 μL of transfection reagent and 4 μL of the BACE1-AS siRNA. Two milliliters of the transfection mixture or the control solution were added to each well. After 6 h, the medium was replaced with 10% fetal bovine serum and incubated for 44 h.

### Stereotactic injection of the mouse hippocampus and sampling of the brain tissues

All procedures described in this study were performed in accordance with Directive 2010/63/EU in Europe and approved by the Ethical Committee for Animal Experiments of Shandong University. Three-month-old C57BL/6J mice (Purchased from Beijing HFK Bio-Technology Co., Beijing, China.) were randomly divided into two groups of 7 mice each: the experimental group and the control group. A 2 μL solution containing 1 μg of Aβ1-42 (Sigma, San Francisco, California, USA) was injected into the CA1 region of the left hippocampus in the mice in the experimental group using a stereotactic technique. The mouse was anesthetized by an intraperitoneal injection of 10% chloral hydrate and fixed on a ZH-LAN-STAR B mouse brain stereotaxic apparatus. The anterior fontanelle was exposed through a top middle incision. According to the vertical position in mouse brain atlas, the needle of a microsyringe was inserted vertically 2.5 mm from the brain surface, 1.6 mm behind the anterior fontanelle and 1.7 mm on the left side of the midline. The injection was performed over 3 min, and the retention time was 2 min. Then, the needle was slowly withdrawn (1 mm/min). All operations were performed under sterile conditions. The skin incision was sterilized with penicillin and the wound was sutured. An equal amount of PBS was injected into the same region of mice in the control group.

Three days after the injection of Aβ1-42, the expression of BACE1-AS and BACE1 mRNA, and the Aβ1-40 contents were detected. The mouse was euthanized by cervical dislocation. The animal was decapitated and the skin on top of the skull was quickly removed, exposing the anterior–posterior fontanelle. A small hole was generated in the front and rear fontanel fontanelle with the tips of small scissors to completely expose the mouse brain. The mouse brain was removed and rapidly frozen in liquid nitrogen.

### RNA extraction from cells and brain tissues

Total RNA was extracted from cultured cells and mouse brains and purified with an RNeasy Midi kit (Qiagen, Hilden, Germany) according to the protocol provided by the manufacturer. RNA integrity was assessed using the Agilent 2100 BioAnalyzer (Agilent, Santa Clara, CA). The RNA concentration was calculated using the following formula: RNA concentration (g/L) = OD260 * dilution factor * 40/1000 (g/L).

### Fluorescence qPCR detection using the TaqMan probe

TaqMan PCR assays for every gene were performed in duplicate using cDNA samples in 384-well optical plates with an ABI Prism 7900 Sequence Detection system (Applied Biosystems). For each 5 μL TaqMan reaction, 2.5 μL of the cDNA templates (1:100 dilution, which corresponds to approximately the amount of cDNAs produced from 12.5 ng of RNA) was mixed with 0.25 μL of 20× TaqMan Gene Expression Assays and 2.25 μL of 2× TaqMan Universal PCR Master Mix (Applied Biosystems). Parallel assays were performed for each sample with the appropriate primers (see Table 1 for primer sequences). Standard curves were prepared for every gene analyzed using serial dilutions of a control entorhinal tissue. Finally, all TaqMan PCR data were analyzed using the Sequence Detector Software version 1.9 (Applied Biosystems).

### Extraction of cell and total proteins from brain tissues

Cultured cells were collected in 6 mm petri dish by digestion and centrifuged at room temperature, and the pellet was prepared as a single cell suspension with 1 mL of 1× PBS and placed on ice. According to the abundance of the target protein and the total number of cells, an appropriate amount of protein lysis solution (ice manipulation) was added. The cells were rapidly and completely lysed after trituration and denatured at 100 °C for 5 h. A 5 μL aliquot was used to quantify the protein concentration and the remaining sample was stored at − 20 °C.

Brain tissue samples were snap frozen and stored at − 80 °C for future experiments. Brain samples were homogenized in ice-cold RIPA lysis buffer (Beyotime, Shanghai, China). The homogenized samples were centrifuged at 12,000*g* for 20 min at 4 °C. The supernatant was collected for Western blotting.

### Western blot analysis

Proteins were separated on SDS-PAGE gels and transferred to PVDF membranes. Membranes were blocked with 5% nonfat dry milk in TBST for 1 h and incubated with primary antibodies overnight at 4 °C. The following primary antibodies were used: BACE1 (Abcam), CTF (Abcam), and β-actin (Santa Cruz). The results were scanned and analyzed using the QUANTITY ONE (v4.6.2) gel electrophoresis image analysis software.

### ELISA

The hippocampal tissues from the mouse brain or supernatants from cultured cells were isolated. Hemibrains were flash frozen and stored at –80 °C until homogenization for Aβ measurements. Aβ1-40 and Aβ1-42 enzyme-linked immunosorbent assays (ELISAs) were performed using ELISA kits (Invitrogen, USA). Aβ standards (Aβ1-40 or Aβ1-42) were prepared according to the manufacturer’s manual. One hemisphere was homogenized in 8 volumes of ice-cold guanidine buffer (5.0 M guanidine-HCl and 50 mM Tris–HCl, pH 8.0) (6 mice per group). The homogenate was incubated at room temperature for 4 h and diluted 1:20 with ice-cold BSAT-DPBS reaction buffer (Dulbecco’s phosphate-buffered saline containing 5% BSA, 0.03% Tween-20, 0.2 g/L KCl, 0.2 g/L KH2PO4, 8.0 g/L NaCl, and 1.150 g/L Na2HPO4, pH 7.4) containing 1 × protease inhibitor cocktail (PMSF, aprotinin, leupeptin, EDTA, pepstatin A, NaF, and NaVO3). Samples were then centrifuged at 15,000×*g* for 20 min at 4 °C. The supernatant was used for the Aβ ELISA.

### Data and statistical analysis

At least three independent experiments were conducted for t tests or variance analyses. The data were analyzed using the SPSS statistical package (SPSS, version 13.0). Values presented are mean ± se. P-values of ≤ 0.05 were considered significant. Asterisks in figures denote statistically significant differences; differences without marks are considered nonsignificant.

## Data Availability

The datasets used and/or analysed during the current study available from the corresponding author on reasonable request.
